# A Mismanaged Memorial

**Published:** 1895-09-14

**Authors:** 


### The Hospital

AN INSTITUTIONAL JOURNAL OF

The Medical Sciences and Hospital Administration,

WITH

AN EXTRA NURSING SUPPLEMENT.

VOL. XVIII.—No. 467.] [SEPT. 14, 1895.

### A MISMANAGED MEMORIAL.

THERE are few lessons harder to learn and yet better worth the learning than that of the relatively small importance of the life of any man, however great, to his fellows. No one is, in fact, indispensable. The accuracy of the cynic's definition of gratitude is confirmed by practice in each day's experience of life. A lively expectation of favours to come seems a caricature of gratitude, but no sooner is a man dead than the attitude of men towards himself and his memory changes utterly. No more striking or painful example of this fact has happened in the present century than the proceedings in connection with the memorial to the late Sir Andrew Clark, Bart., M.D., F.R.S. At the moment of his death high and low combined to praise the man and to express a desire to perpetuate his memory. In the Press and from the pulpit eulogies were delivered which justly recorded the measure of affection and appreciation which a multitude felt for the lamented physician, his character, and his work. The Duke of Cambridge placed himself at the head of a movement to raise a fitting testimonial, and Mr. Gladstone came from his sick-room at no little risk to himself, in order to testify to his appreciation and affection for the dead friend and medical adviser.

It was determined by the committee charged with the arrangements to raise £12,000 in order to defray the cost of erecting a block of buildings at the London Hospital, containing an ample number of small wards for the isolation, observation, and treatment of certain cases always liable to obtain admission or to develop themselves under treatment in a general hospital. Such cases, being of an infectious nature, cannot with safety to the main body of patients be treated in the medical wards without danger to others, and if sent away to a fever hospital they are exposed to fresh dangers to themselves. The contemplated buildings were to be so arranged as to contain a complete pathological department, a branch of science with which Sir Andrew Clark was closely identified during the earlier years of his life, when he held office as assistant physician at the London Hospital, and to the study of which he attached the greatest importance, as was constantly shown in his medical teaching.

At the time of the meeting in May, 1894, when Mr. Gladstone spoke in Princes' Hall, nearly £3,000 had been raised. It will be in the remembrance of all that the meeting was crowded with influential, wealthy, and representative personages. Nothing more touching or better calculated to appeal to the hearts of all who had reason, either as friends or patients, for gratitude or regard for Sir Andrew Clark could have been uttered than the eloquent speech which Mr. Gladstone delivered. Alas! for poor human nature, for at the meeting of the quarterly court of governors of the London Hospital, held a week ago, it was reported that the appeals for funds to worthily perpetuate the memory of the late Sir Andrew Clark had not met with so generous a response as was anticipated, and that the total amount subscribed barely reached £3,000. In other words, so little use has been made of the interest excited by Mr. Gladstone's speech that it has produced practically no additional subscriptions. In these circumstances those responsible—it was not mentioned with whom the decision rested, although we think so impotent a decision should be placed on the right shoulders—have abandoned the form of memorial set forth above, and have "substituted an erysipelas ward for women, another for isolation cases, and increased accommodation for the porters." Surely impotent is not too strong a term to apply to such a conclusion, especially when Mr. Gladstone's words are remembered. For Mr. Gladstone rightly stated that the Duke of Cambridge had never given his countenance "to a worthier cause than the memorial to Sir Andrew Clark," which associated itself with the profession at large, for a man's "great devotion to the purpose of the profession was never exemplified in a more remarkable manner than in the career of the late President of the Royal College of Physicians." Mr. Gladstone added: "I think the profession, if I may presume to say so, has done well in determining that Sir Andrew Clark might in the present age be taken up by common consent as a typical man, representative of all that is best and noblest in the profession and in its purposes." These burning words were recognised as just and true when they were uttered, and we believe the decision we have referred to will cause a feeling of widespread indignation, not only in the profession of medicine, but in all professions, for there is none which does not contain very many members who have a recollection of the debt they owe to the skill, kindness, and attention of the late Sir Andrew Clark.

This is, of course, a very delicate matter to deal with in the Press, but there are occasions when plain speaking is called for, and we feel that the present is one of them. If those responsible for the decision to erect a building for the accommodation of the male servants of the London Hospital as a memorial to Sir Andrew Clark, whoever they may be, do not feel heartily ashamed of themselves when they come to think the matter over, we shall be sorry for them. In our view, as an alternative, it would be far better to call the testimonial committee together to pass a resolution recording the committee's incapacity to raise a suitable memorial, and to return the money they have received, rather than to do this grievous wrong to the memory of one of the noblest and, on the whole, one of the greatest and most statesmanlike members that the medical profession has ever had. In any case, the idea of memorialising the late Sir Andrew Clark by the erection of a servants' wing to the London Hospital must be abandoned. Three thousand pounds, it is true, is not a large sum, but it is large enough to secure, if an inadequate, still a suitable memorial befitting the occasion, the man, and his work.

The whole proceedings in connection with this movement must tend to deepen the feeling which animates most men of reputation. This feeling leads them to refuse to permit any testimonial of any kind to be raised to them during their lifetime. We are inclined to think that the unhappy proceedings in Sir Andrew Clark's case may even call forth precautions for the protection of the dead. Many men now living may well decide to introduce a clause into their wills forbidding any step being taken to raise a memorial to themselves or their work after their decease. An inadequate memorial is liable to lead to the inference that the object of it was not after all a *persona grata* with his contemporaries. In the present instance, as Mr. Gladstone proved, anything more unjust or untrue it would be difficult to mention, but the sting of it must unfortunately remain, to the lasting condemnation of those who are immediately to blame.

